# The Real Era of the Art of Medicine Begins with Artificial Intelligence

**DOI:** 10.2196/16295

**Published:** 2019-11-18

**Authors:** Bertalan Meskó

**Affiliations:** 1 The Medical Futurist Institute Budapest Hungary

**Keywords:** future, artificial intelligence, digital health, technology, art of medicine

## Abstract

Physicians have been performing the art of medicine for hundreds of years, and since the ancient era, patients have turned to physicians for help, advice, and cures. When the fathers of medicine started writing down their experience, knowledge, and observations, treating medical conditions became a structured process, with textbooks and professors sharing their methods over generations. After evidence-based medicine was established as the new form of medical science, the art and science of medicine had to be connected. As a result, by the end of the 20th century, health care had become highly dependent on technology. From electronic medical records, telemedicine, three-dimensional printing, algorithms, and sensors, technology has started to influence medical decisions and the lives of patients. While digital health technologies might be considered a threat to the art of medicine, I argue that advanced technologies, such as artificial intelligence, will initiate the real era of the art of medicine. Through the use of reinforcement learning, artificial intelligence could become the stethoscope of the 21st century. If we embrace these tools, the real art of medicine will begin now with the era of artificial intelligence.

## From the Dawn of Medicine to the 21st Century

Since Hippocrates, physicians have been performing the art of medicine. With limited knowledge, experience, and rudimentary tools, they have been the guardians of health and disease. The medical profession is highly regarded and has always been considered exceptional, and since the ancient era, patients have turned to physicians for help, advice, and cures. When the fathers of medicine started writing down their experience, knowledge, and observations, treating medical conditions became a structured process with textbooks and professors sharing their methods over generations.

In his essay “Teacher and Student”, Sir William Osler noted that: “The practice of medicine is an art, based on science,” [1]. Physicians had to possess the necessary skills to make use of their vast knowledge and experience, but they also had to have a spiritual understanding of treating the patient and not the disease [2]. Some argue that it is the mission of medical professionals to provide the art and science of medicine [3]. Then, at the dawn of modern medicine in the late 19th and early 20th century, there were attempts to transform the art of medicine into science to supplement clinical judgment using the conclusions of a scheme of logic [4]. After evidence-based medicine was established as the new form of medical science, it seemed that the art and science of medicine had to be connected [5]. Physicians were expected to treat the patient and not the disease, had to deal with a myriad of data about their patients and had to handle the increasing availability of new clinical science and new studies.

In the case of the first stethoscope, a hollow wooden tube introduced by Dr. Laennec in France in the early 19th century to augment cardiac and lung sounds, it took decades to spread the idea of improving care with it [6]. That one innovation needed so much time to reach the masses and become a common medical practice as physicians were reluctant to use it. Now dozens of innovations come out daily, and the burden and pressure are enormous on physicians to adopt them properly.

By the mid-20th century, health care had become highly dependent on technology. Physicians had to learn to work closely with it, which is consequentially a major contributor to their burnout today [7]. When personal computers became widely available, the concept of electronic health emerged. The digitalization of medical records became inevitable, as the amount of available medical knowledge continued to grow rapidly by millions of new studies every year, and patients started to become empowered to oversee their own health care. When personal computers could be connected to networks, telemedical services appeared. The rise of social media networks gave space for the rise of Medicine 2.0 and Health 2.0, while the penetration of mobile phones and later smartphones resulted in mobile health. However, since the 2010s, the rate at which disruptive technologies appear has become overwhelming for both patients and their caregivers [8]. This is the cultural transformation that, under the term digital health, has been shaping the fundamental structures of medicine and the doctor-patient relationship.

The doctor-patient hierarchy has been transforming into a partnership, with many formerly passive patients becoming proactive in their care and wanting to be involved in decision making. The ivory tower of medicine has been breaking down and opening for nonprofessionals too. Thus, digital health technologies could be considered a threat to the art of medicine [9], but I argue that it is not the case. On the contrary, advanced technologies and proactive patients will initiate the real era of the art of medicine.

## Artificial Intelligence Will Facilitate the Rise of the Art of Medicine

Despite common fears about the perceived dangers of automation, especially artificial intelligence, it does not seem to be taking the jobs of physicians or monopolizing medicine. As studies show, it will instead help automate administrative tasks and take over monotonous day-to-day assignments [10]. It has the potential to free up time for medical professionals to let them fulfill the mission they signed up for: to help people on their health care journeys with compassion, creativity, and care.

The art of medicine requires attention, time, and empathy from physicians while they are treating patients [11]. With the challenging interfaces of electronic medical records, a lot of administrative tasks that need to be handled, and a constant pressure from payers impacting physicians, even the chance for the art of medicine vanishes. However, the use of artificial intelligence (AI) could help facilitate the art of medicine [12].

Advanced technologies such as AI will transform the role of physicians [13]. Medical professionals might prefer to be the translator of the technical data for the patient, act as a guide in the jungle of digital health for their patient, and be a counselor in navigating through health care choices, instead of being the ultimate source of medical knowledge and the sole decision-maker regarding medication and treatment choice. There are case studies that underscore this notion [14]. There was a game between the reigning chess world champion Garry Kasparov and IBM’s Deep Blue supercomputer in 1997 that ended in a triumph for the algorithms/the programmers of the algorithm. It was believed then that this win might indicate the end of chess, because why would anyone play chess when the best player in the world was a program? However, over 600,000 million people play chess today, which is higher than in previous years. No professional player can prepare without working with computers now, as technology has made it more popular and increased the level at which players can play.

A similar path awaited the Chinese game, Go, which is one of the most complex games ever invented. In chess, the number of moves at any given moment in a game is approximately thirty. On the 19×19 square grid of the Go board, players can have around 200 moves, with the number of configurations on the board more than the number of atoms in the universe. This is only one of the factors that made the victory of Google Deepmind’s AlphaGo over the 18-time Go World Champion, Lee Sedol, a milestone in computation and a spectacle for 80 million viewers worldwide [15]. Winning in Go requires creativity and intuition, human skills that are beyond current artificial intelligence technology. The key to success for AlphaGo was in the way in which it was trained to play the game [16], as it used a combination of neural networks and reinforcement learning. In reinforcement learning, developers don’t tell the algorithm how to play the game and instead let it play millions of games against itself, only telling the algorithm which outcomes were the desired ones (the matches the algorithm could win) and letting the program find its strategy and rules for how to achieve those desired outcomes. This means that the cognitive limitations of people are not programmed into the algorithm. In theory, it could find new ways of playing the game even though it has been played by millions of people for thousands of years. Thus, such algorithms are not affected by the concepts, philosophies, beliefs, and mechanisms of human thinking, nor are they affected by the boundaries and flaws of human intelligence.

Advanced algorithms have already been shown to improve several aspects of practicing medicine [17], including radiology, oncology, cardiology, medical errors, and cost-effectiveness, among others. An algorithm could use reinforcement learning to find a treatment or even a cure for a medical condition that physicians and researchers cannot, and while the treatment or cure works, the underlying reasons why are not clear. Patients get better, get cured, or can manage their condition properly, but the way it was found is not clear or understood by those who administer the therapy. This would be the era of the modern art of medicine. Medical professionals using advanced technologies can care for their patients better, and their challenge is not in finding out how to care for their patients but instead to understand why certain methods work and others do not. When clinical science is not lagging behind the practice of medicine but progressing ahead of it, the art of medicine is going to be understanding how algorithms got to a conclusion.

Another way that AI could help physicians practice the art of medicine is if watched over these algorithms and evaluated outputs against their best clinical judgment as a safety check. Systems will inevitably get the answer wrong occasionally, and we will need good clinical judgment to intervene when that occurs.

## Preparing for the Modern Art of Medicine

AlphaGo was able to beat the world champion four to one. Lee Sedol was deeply disappointed and felt embarrassed at first, as he felt he was representing humans in a losing battle against a technological entity. However, by the fifth game they played, it seemed that Sedol had a newfound understanding of the way AlphaGo evaluated the game and its positions. He stated that by doing that he became a much better player. He was able to stand on the shoulders of technology to further develop his skills and vision about the future of the game. This is exactly how the medical community should look at the impact digital health technologies have on health care and on their jobs.

In the next decade, it is believed that most patients will become empowered and proactive in their care, using sensors to obtain data, and using the information they find online as well as algorithms to understand this health data. Seamless interfaces and invisible technologies, such as chatbots with voice-to-text functions and algorithms scanning the medical literature, will become a common element of practicing medicine. Moreover, artificial narrow intelligence algorithms will discover new treatments and run *in silico* clinical trials that physicians, pharma companies, or medical innovators would never think of. As it will not be limited by the traditional pathways and thought patterns used for centuries in medicine, artificial intelligence may produce entirely new solutions for tackling global health issues.

The supercomputer in Douglas Adam’s “The Hitchhiker’s Guide To The Galaxy”, after thinking for seven and a half million years, provided the answer to life, to the universe, and everything: 42 [18]. What if smart algorithms just started spitting out the answers to big questions without any explanation? The real art of medicine would thus be figuring out the logical path of how an AI arrived at a certain solution. That will require high levels of creativity, problem-solving, and cognitive skills from the medical community.

**Figure 1:**
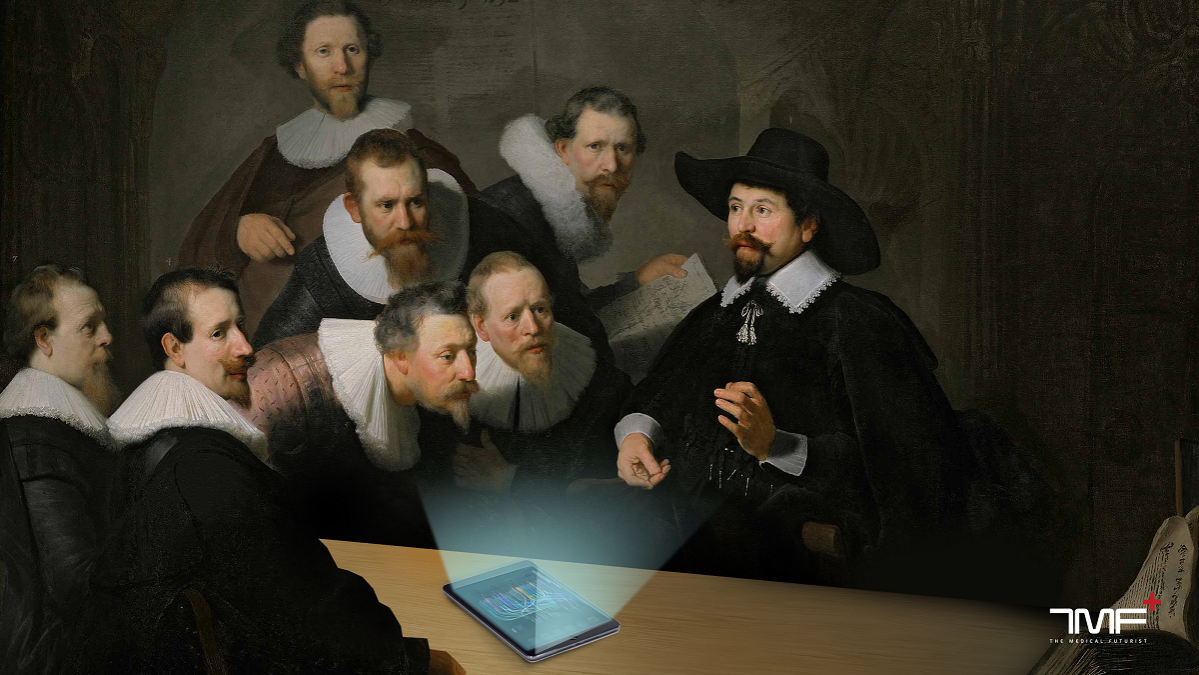
A concept art about the art of medicine in the era of artificial intelligence based on Rembrandt's painting The Anatomy Lesson of Dr Nicolaes Tulp.

I believe that AI is going to be the stethoscope of the 21st century. While digital health technologies provide us with more health data than ever before and AI helps analyze it to improve health and well-being, to cut down on administrative tasks, or even to optimize both physicians’ and patients’ schedules, we should never forget that they are going to be tools in the hands of physicians and not the other way around [19]. We can be confident that compassionate care, empathy, creativity, problem-solving, and profound human connection will never cease to be at the core of caring for patients. If we embrace these tools through well-designed policies, constant education, and proper guidelines, the real art of medicine will begin now with the era of artificial intelligence (Figure 1).

## Abbreviations

AI: artificial intelligence
